# Effects of Berberine on Atherosclerosis

**DOI:** 10.3389/fphar.2021.764175

**Published:** 2021-11-26

**Authors:** Rui Rui, Haolan Yang, Yanke Liu, Yue Zhou, Xudong Xu, Chaohong Li, Shuying Liu

**Affiliations:** Department of Histology and Embryology, Zhongshan School of Medicine, Sun Yat-sen University, Guangzhou, China

**Keywords:** berberine, atherosclerosis, endothelial cell, vascular smooth muscle cell, inflammatory cell, vascular stem cell

## Abstract

Atherosclerosis is an epidemic across the globe[A1], and its morbidity and mortality remain high, involving various complications and poor prognoses. In atherosclerosis, endothelial cells (ECs) dysfunction, vascular smooth muscle cells (VSMCs) migration and proliferation, foam cell formation, and inflammatory cell recruitment contribute to disease progression. Vascular stem cells (VSCs) also play a critical role in the cardiovascular system. Important data showed that the simultaneous increase of proliferation and apoptosis of VSMCs is the main cause of graft vein stenosis, suggesting that inhibition of VSMCs proliferation and apoptosis simultaneously is an important strategy for the treatment of atherosclerotic stenosis. Complementary and alternative medicine use among patients with cardiovascular disease (CVD) is growing. Berberine is an extract of Chinese traditional herbs that is known for its antimicrobial and anti-inflammatory effects in the digestive system. Its underlying anti-CVD mechanisms are currently attracting interest, and its pharmacological actions, such as antioxidation, regulation of neurotransmitters and enzymes, and cholesterol-lowering effects, have been substantiated. Recent studying found that berberine could inhibit both the proliferation and apoptosis of VSMCs induced by mechanical stretch stress simultaneously, which suggests that berberine might be an excellent drug to treat atherosclerosis. This review will focus on the recent progress of the effect of berberine on vascular cells, especially VSMCs, to provide important data and a new perspective for the application of berberine in anti-atherosclerosis.

## Background

**FIGURE 1:**
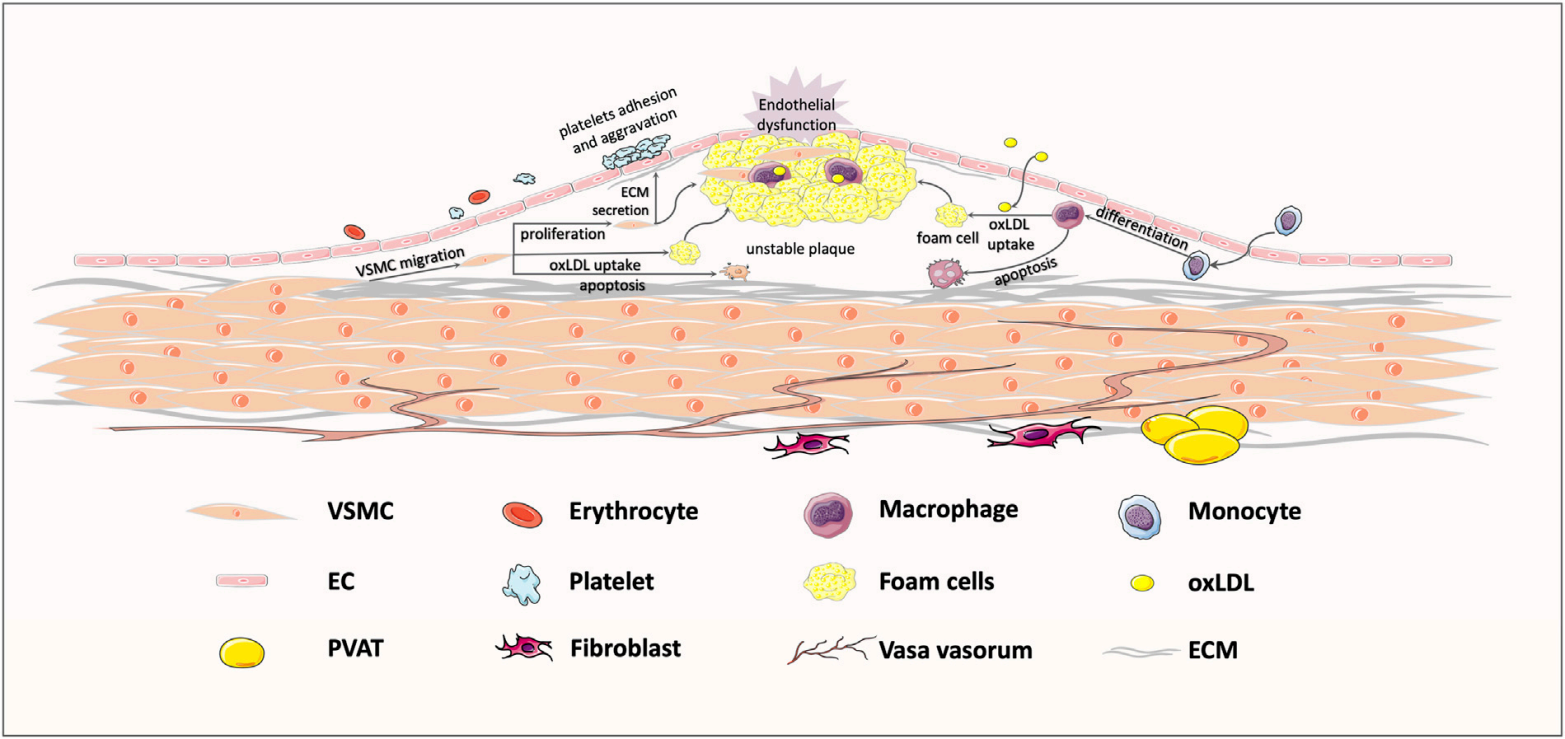
Typical response to vessel injury, in an atherosclerosis example. Exposure to some risk factors, like hypertension, hyperglycemia, and hyperlipemia, might bring about endothelial injury and subsequent lipoprotein infiltration. The infiltrating low-density lipoprotein (LDL) is oxidized to oxLDL, which could be recognized by transmembrane receptors. Then, macrophages and VSMCs migrate to the intima to take up oxLDL, shifting the cell phenotype to foam cells. Foam cell recruitment and vascular smooth muscle cells (VSMCs) proliferation jointly contribute to the formation of unstable plaques, ultimately leading to vessel stenosis.

Cardiovascular disease (CVD) has reached pandemic proportions, and clinical treatment remains problematic. Atherosclerosis generally involves the large arteries, which include three heterogeneous layers; from inside to out, they are the tunica intima, the tunica media, and the tunica adventitia. The layers are organized by different types of cells, such as endothelial cells (ECs), vascular smooth muscle cells (VSMCs), and inflammatory cells, that play individualized roles (Stenmark et al., 2013). Studies have also demonstrated that vascular stem cells (VSCs) differentiate into myofibroblasts and migrate to the intimal sites to accelerate neointimal hyperplasia in response to injury, and this process is an independent contributor in CVD (Kawabe and Hasebe, 2014). In normal physiological conditions, the vascular cells are organized according to their functions, and they secrete various vasoactive substances to regulate vascular physiological activities (Table 1) (Qiao et al., 2010; Wang D. et al., 2017). However, some pathological factors, such as mechanical stretch stress, oxidized low-density lipoprotein (oxLDL), and advanced glycation end products (AGEs), might lead to an imbalance of vascular microenvironment homeostasis, resulting in hemodynamic changes and vascular remodeling. For instance, calcium and phosphorus deposition generally occurs within either the tunica intima or media and leads to endothelial injury (Wu et al., 2013). The thickened tunica media associated with VSMC proliferation usually causes stenosis of arteries (Bennett et al., 2016). Inflammation often occurs in the tunica adventitia because of the connective tissue and a special type of adipose tissue, perivascular adipose tissue, that surrounds it (Tinajero and Gotlieb, 2020). The presence of every condition identifies individuals at greater risk for CVD events (Figure 1); among the conditions, hypertension-induced mechanical stress is a significant factor in atherosclerosis.

**TABLE 1:** Vasoactive substance synthesized by cells in the arterial wall.

Sites	Vasoactive substance	Main functions
Endothelial cells	NO	Vasodilation, reducing myocardial contractility and inhibiting platelet adhesion and aggregation
	PGI2	Vasodilation and inhibiting the clotting process
	Adenosine	Cooperating with NO to exert vasodilator effect
	ET	Vasoconstriction and promoting the proliferation of VSMCs in tunica media
	TXA2	Vasoconstriction and promoting platelet aggregation
Vascular Smooth Muscle	H_2_S	Vasodilation, reducing myocardial contractility, inhibiting vascular remodeling, and protecting the myocardium
	ET	Vasoconstriction and promoting the proliferation of tunica media
	AngII	Vasoconstriction
Adipocytes in Tunica Adventitia	PVRF	Vasodilation

NO, nitric oxide; PGI2, Prostacyclin; ET, Endothelin; TXA2, Thromboxane A2; AngII, AngiotensinII; PVRF, PVAT-derived relaxing factors.

Vascular stenosis caused by thickening of vessel walls remains an intractable problem, mainly because of damage associated with hypertension. Treatment of atherosclerosis contains three stages. The early stage relies on drug therapy; stent implantation is the main treatment in the middle stage; and vascular transplantation is considered for the final stage. Although drug therapy has shown some effects, many patients experience progression into the middle stage of atherosclerosis treatment. Stent implantation is common, but postoperative vascular restenosis is an inescapable problem despite improvements to vascular coating technology and the use of degradable stents. As a result, many patients will eventually undergo advanced treatment for atherosclerosis, requiring the coronary bridging surgery of vascular transplantation because of complete stenosis and closure of the vessels caused by the atherosclerotic plaques. Thus, finding a new drug to treat atherosclerosis would be urgent.

Statins are widely used as the first-line drugs to treat atherosclerosis because of their excellent lipid-lowering functions. However, some patients treated with statins do not achieve the expected efficacy and might have side effects, such as blood glucose changes and hepatotoxicity. For these patients who experience unsatisfactory lipid-lowering effects, new alternatives are urgently needed. As the complementary and alternative medicine (CAM) has draw more attention, in recent years, interest in the role of herbal plants to treat CVD has grown. *Allium sativum* (garlic), *Andrographis paniculata*, and other herbal plants have demonstrated antihypertensive effects (Rastogi et al., 2016). Berberine [5, 6-dihydro-9, 10-dimethoxybenzo(g)-1, 3-benzodioxolo(5,6-a) quinolizinium, or C_20_H_18_NO_4_], a benzylisoquinoline alkaloid isolated from several Chinese herbal substances, has been widely used in the treatment of inflammatory disorders, microbial and protozoal infections, and intestinal diseases in Ayurvedic medicine and in traditional Chinese medicine for centuries. One study has shown that berberine and its derivatives definitely lower blood glucose, blood lipids, blood pressure, anti-oxidative stress, and atherosclerosis, and they play a medicinal role in CVD and metabolic disorders, such as atherosclerosis, heart failure, myocardial infarction, stroke, nonalcoholic fatty liver diseases, and diabetic cardiomyopathy (Feng et al., 2019). As an antibacterial drug, berberine has been used in clinical practice for many years with few side effects and a low price that greatly reduces the economic burden on patients. One study found that the combination of simvastatin and berberine did not increase the side effects of drugs (Li et al., 2019); another study showed that the blood concentration of each drug increased when given in combination (Liu M. et al., 2015), increasing the lipid-lowering effect synergistically (Kong et al., 2008). Studying berberine as a treatment for CVD is particularly important for patients who are not ideally suited for conventional treatment, such as statins. In a network pharmacology investigation, 31 and 331 putative targets for berberine and atherosclerosis, respectively, were identified (e.g., the MAPK and PI3K-Akt signaling pathway) and provide valid evidence for the curative effects of berberine on atherosclerosis (Xie et al., 2020).

**FIGURE 2:**
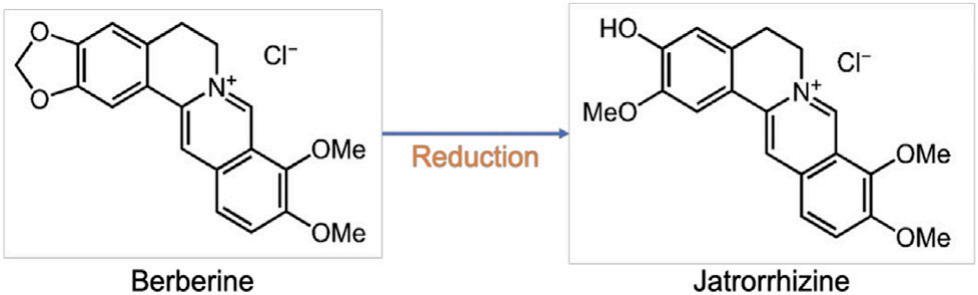
Structures of berberine and jatrorrhizine.

Berberine and its metabolites have biological activities *in vivo*. For example, jatrorrhizine, identified as a hydrogenation metabolite of berberine in humans, exhibits powerful antiradical and antioxidant activities resulting from its free phenolic group (Figure 2) (Wang K. et al., 2017). Although the bioavailability of berberine is less than 1% according to pharmacodynamic experiments, berberine and its metabolites have high tissue distribution (Tan et al., 2013), and research has shown that berberine carried by liposomes has higher bioavailability and selectivity than berberine oral administration (Lagoa et al., 2020). Despite its extremely low plasma concentration, berberine conveys significant pharmacological actions against CVD. Several studies have demonstrated the therapeutic effects of berberine on multiple vessel cells in CVD and will be discussed in *Berberine Inhibits EC Dysfunction* through *Effect of Berberine on VSCs* sections.

## Berberine Inhibits ECs Dysfunction

As the most inner layer of the vasculature, the endothelium is critical for normal vascular function. It serves as a physiological barrier between blood and tissues. Normally, the endothelium is smooth and intact, helping to prevent platelet adhesion.

ECs exhibit antiproliferative and anti-inflammatory functions and maintain hemostatic balance. These cells also synthesize and release various beneficial substances (Table 1), such as nitric oxide (NO), Prostacyclin, and Endothelium-derived hyperpolarizing factor, which help tissues take in oxygen and help vessels keep a balanced state, inhibiting endothelial dysfunction (Marti et al., 2012).

### ECs Function in the Atherosclerosis

Vascular endothelial dysfunction is an early event in atherosclerosis. The inflammatory response is accompanied by endothelial cell dysfunction (ECD). High oxidative stress and reduced NO availability are the main causes of ECD, which increases vascular remodeling (Hansson, 2005).

ECD is initiated by multiple inflammatory factors in atherosclerosis; when the endothelium is injured, inflammatory mediators, including vascular cell adhesion molecule-1 (VCAM-1), intercellular adhesion molecule-1 (ICAM-1), interleukin 6 (IL-6), and tumor necrosis factor α (TNF-α)are expressed. The expression activates oxidative stress and the inflammatory response to induce VSMCs proliferation and foam cells formation, which lead to plaque formation and, eventually, atherosclerotic lesions. OxLDL is also a major contributor to ECD. It is deposited in the vascular endothelium and becomes involved in plaque formation (Tan et al., 2020). Increasing amounts of xanthine and NADH/NADPH oxidases as well as reduced endothelial NO synthase (eNOS) are important responses to high oxidative stress in ECs. Normally, eNOS produces NO in the vessel endothelium; in the inflammatory process, inducible NO synthase (iNOS) expresses in macrophages, and smooth muscle cells produce NO in a process closely related to reactive oxygen species (ROS) production. ROS are oxygen byproducts of cellular metabolic reactions, including superoxide anion (O_2_^−^), hydrogen peroxide (H_2_O_2_), and the hydroxyl radical (HO^•^). ROS play the roles of secondary messengers in signaling pathways that underlie key events, such as cell differentiation, growth, and death (Cheng et al., 2017). Berberine also contributes to amelioration in endothelial dysfunction and vascular inflammation.

### Berberine Protects ECs From Damage

Berberine improves endothelial function. Elevated endothelial microparticles (EMPs) in the circulation, mostly defined as CD31^+^/CD42^−^ microparticles, could serve as markers of ECD and arterial stiffness. EMPs are also closely related to vascular dysfunction in cardiovascular disorders. Berberine may help decrease circulating EMPs. In human umbilical vein ECs (HUVECs), berberine treatment in healthy volunteers decreased circulating CD31^+^/CD42^−^ microparticles and improved flow-mediated vasodilation. In spontaneously hypertensive rats (SHRs), berberine treatment partly reduced the blood pressure and circulating EMPs. In addition, berberine preserved arterial elasticity by increasing the content of arterial media elastin fiber and improved endothelial function by maintaining better endothelium-dependent vasodilation (Zhang G. et al., 2020). In ECs exposed to microparticles, berberine prevented microparticle-induced eNOS downregulation and maintained NO formation to stop EMPs from damaging ECs function (Wang et al., 2009).

Many substances, including visfatin, oxLDL, and multiple inflammatory factors, serve as mediators in the pathways related to ECD, and berberine plays a suppressive role to these stimuli. Visfatin, also known as pre-B cell colony enhancing factor, and nicotinamide phosphoribosyl-transferase could react to many inflammatory cytokines like IL-6 and TNF-α. Visfatin activates inflammation and cholesterol accumulation by modulating the expression of scavenger receptors (SR)-A and CD36, leading to ECD and resulting in atherosclerosis. In apolipoprotein E-knockout (ApoE^−/−^) mice, oral administration of berberine (5 mg/kg/day orally for 12 weeks) lowered the serum levels of visfatin and inflammatory cytokines and reduced the level of visfatin in the atherosclerotic plaques (Zhou et al., 2013). Another study indicated that berberine suppressed visfatin-induced apoptosis of HUVECs by inhibiting the p38 MAPK and JNK signaling pathways, suggesting a therapeutic effect in atherosclerosis (Wan et al., 2018). In another study, berberine also protected HUVECs from lipopolysaccharide-induced injury by blocking activation of the JNK pathway, which subsequently promoted the expression of the antiapoptotic protein myeloid cell leukemia-1 (Guo et al., 2016).

The nuclear factor kappa-B (NF-κB) signaling pathway is also involved in ECs apoptosis and turnover that ultimately contributes to the formation of atherosclerotic plaques. Inflammatory stimuli, such as oxLDL and TNF-α, could activate the ROS/NF-κB signaling pathway and increase the expression of LDL receptor-1 (LOX-1), leading to endothelial injury. LOX-1 activation also promotes ROS production, indicating a positive feedback loop between ROS and LOX-1. Berberine significantly decreased the TNF-α–induced expression of transcription factors p52 and p65 in the NF-κB signaling pathway, which could be a possible mechanism of reducing LOX-1 expression by berberine. Berberine also attenuates NF-κB by activating AMPK. In addition, berberine could protect HUVECs against oxLDL-induced injury and apoptosis by inhibiting mitochondrial membrane potential collapse, chromosome condensation, cytochrome-C release, and caspase-3 activation (Hsieh et al., 2007). Berberine alleviated atherosclerotic lesions in ApoE^−/−^ mice fed a Western-type diet for 12 weeks by reducing serum lipid levels, antagonizing hepatic lipid accumulation, and improving intima-media thickening. Meanwhile, berberine also reduced aortic ROS generation and the serum levels of malondialdehyde, oxLDL, and IL-6. In an aortic ring assay, berberine restored aortic endothelium-dependent vasodilatation *in vivo* and *in vitro* (Tan et al., 2020). These results indicate the medicinal effects of berberine against atherosclerosis and hypercholesterolemia.

NO suppresses endothelial inflammation and adhesion to inhibit thrombosis and promotes angiogenesis cooperatively with growth factors in microvessels. Berberine regulates the eNOS/NO balance and directly helps ECs release NO to ameliorate vasoconstriction. A study showed that berberine increased NO and cGMP production to induce both endothelium-dependent and endothelium-independent relaxation in rat aortic rings, exerting a hypotensive effect (Kang et al., 2002). Endothelium-derived NO production is mediated by the Akt/eNOS signaling pathway (Guo et al., 2008). AMPK also promotes the formation of eNOS and heat shock protein 90 complex through phosphorylation of the eNOS serine 1177 site (Morrow et al., 2003). Berberine treatment upregulated the expression levels of AMPK and phosphorylated AMPK (p-AMPK) protein in HUVECs cultured with palmitic acid but showed no effect on the expression of Akt and p-Akt protein. These results suggest that AMPK activation may be one pathway used by berberine to regulate NO generation.

Oxidative stress is an important part of ECD. Activation of NADPH oxidase (NOX) could promote ROS formation. In ECs, the main form of expressed NOX is the NOX4 subtype, which is also the main source of oxygen production. EMPs could increase NADPH oxidase activity. Berberine has suppressed the overexpression of NOX2 and NOX4 and has decreased ROS production in endothelial cells. Berberine also appeared to protect endothelial cells against EMPs (Yousefian et al., 2019). AMPK is also regarded as an important inhibitor of NOX in vascular ECs. In HUVECs, berberine inhibited free fatty acid induced eNOS activation and NOX4-derived ROS accumulation through AMPK activation to exert its protective effects on high blood glucose–induced endothelial vasodilation injury. One possible mechanism of berberine-induced AMPK activation is increasing the AMP/ATP proportion (Xu et al., 2017). However, an exact mechanism of AMPK activation by berberine remains to be elucidated.

Endothelium-dependent contraction (EDC) is also associated with endothelial dysfunction. Berberine suppressed cyclooxygenase-2 (COX-2) expression and ROS production, restrained endoplasmic reticulum (ER) stress, and activated AMPK to inhibit EDC. In SHR carotid arteries, berberine suppressed EDCs by reducing COX-2 expression. Berberine also inhibited eIF2α phosphorylation and ATF3, ATF6, XBP1 expressions to prevent ER stress (Liu L. et al., 2015) and reduced EDCs, likely through activating AMPK and then inhibiting ER stress, scavenging ROS, and downregulating COX-2 (Yang et al., 2010).

In summary, berberine inhibits ECD by increasing eNOS/NO expression, decreasing inflammatory factors and cytokines, and reducing oxidative stress and NOX production in ECs. Some aspects need additional exploration: Regulation of berberine *via* AMPK pathway has been studied in depth, but the specific mechanism of activating AMPK remains unclear. Berberine plays roles in various signaling pathways, but questions remain about specific details: Is there any crosstalk among these pathways? Which one is dominant? Are there any other downstream sites of AMPK at which berberine might work to restrict ECD? In the atherosclerosis process, other biological characteristics of ECs in which berberine may play a role are worth investigating. Whether berberine could protect the tight junction of ECs to keep inflammatory cells from migrating into the subendothelial layer is still unknown. Also, it reported that tumor ECs had abnormal glycolysis and tricarboxylic acid cycle metabolism (Rohlenova et al., 2018); what is the role of ECs metabolism in atherosclerosis? Could berberine improve that role? These questions are intriguing and must be explored.

## Effect of Berberine on VSMCs

VSMCs are highly specialized cells that play a principal role in several diseases, such as atherosclerosis, and hypertension. As the major components of blood vessels, VSMCs control vessel tone and diameter, both of which are key players in the regulation of vascular tension and vascular function. Hypertension-induced stretch stress is a key player in VSMCs proliferation, migration, and apoptosis. Various signaling pathways contribute to smooth muscle cell proliferation and migration (Frismantiene et al., 2018), and stretch stress could nonspecifically activate all transmembrane receptors of VSMCs, resulting in a series of intracellular signal transduction (Li and Xu, 2007). Berberine may inhibit the adverse cellular behavior of VSMCs, thus influencing the progression of CVD.

### VSMCs Play Roles in Atherosclerosis

Increasing proliferation and decreasing apoptosis of VSMCs are considered the main causes of atherosclerosis development. Therefore, a clinical treatment strategy is inhibition of proliferation and promotion of apoptosis of VSMCs. However, recent studies suggest that biological mechanical stress caused by hypertension can concurrently increase both proliferation and apoptosis of VSMCs in the wall of a transplanted vein, leading to stenosis and occlusion of vessel and so aggravating the disease (Ping et al., 2017). Thus, a treatment to inhibit both proliferation and apoptosis of VSMCs is needed. The activated VSMCs execute phagocytic and secretory functions, contributing to the formation of the unstable plaque. Moreover, the phenotype of VSMCs is a key element in the progression of atherosclerotic lesions; when exposed to pathophysiologic conditions such as hypertension-related mechanical stress and ROS, VSMCs switch from a contractile to synthetic phenotype, possessing highly proliferative and migratory capacities (Saleh Al-Shehabi et al., 2016). Therefore, a drug that inhibit the proliferation, apoptosis, migration, and phenotype shifting of VSMCs is necessary.

### Berberine’s Curative Effects on VSMCs

The overwhelming majority of research about berberine is devoted to confirming its therapeutic efforts to prevent restenosis of the lumen caused by abnormal proliferation of VSMCs in post-percutaneous coronary intervention. In a rat model of carotid injury, berberine inhibited AngII (angiotensin-II) and heparin-binding epidermal growth factor–induced VSMCs proliferation and migration by suppressing Akt activation; berberine also improved neointimal formation (Lee S. et al., 2006), which suggests that berberine may be a potent agent to control restenosis after balloon angioplasty. Moreover, when platelet-derived growth factor (PDGF) was released by injured vessels, berberine repressed its stimulated-proliferation effects on VSMCs by activating AMPK/p53/p21^Cip1^ signaling and inactivating the Ras/Rac1 pathway as well as downregulating the expression levels of cyclin D/Cdks and the MEK/ERK-dependent transcription factors Egr-1 and c-Fos (Liang et al., 2006; Liang et al., 2008). Another study showed that berberine could generate G0/G1 phase arrest *via* activation of p27 and p21 to attenuate PDGF-BB(dimer of the B chain of PDGF)–induced proliferation (Liu et al., 2011); berberine also upregulated the peroxisome proliferator-activated receptor (PPARα)–NO signaling pathway to restrain Ang IV-stimulated VSMCs proliferation (Qiu et al., 2017). It could not only motivate PPARα but also promote eNOS to increase beneficial NO production to antagonize Ang IV. NO has a vasodilator function, so nitroglycerin has been used in clinical treatment for many CVDs, such as angina pectoris, congestive heart failure, and myocardial infarction. Berberine administration in rats could suppress protein kinase C-α in VSMCs to reverse nitroglycerin tolerance caused by long-term use (Zhang et al., 2021). All these results suggest that berberine may be latently available to prevent restenosis of arteries by inhibiting VSMCs proliferation.

However, given new findings suggesting that both proliferation and apoptosis of VSMCs play crucial roles in atherosclerosis, future research about VSMCs should explore both roles. In a murine model of diabetic atherosclerosis, both proliferation and apoptosis of VSMCs could be induced by hypertension-induced stretch stress and AGEs alone or together, and berberine could inhibit this progress by decrease PDI (protein disulfide isomerase) expression to prevent vein graft stenosis (Ping et al., 2017). In CVD progression, ER stress plays a vital part in VSMCs proliferation or apoptosis (Hetz, 2012). Berberine safeguards VSMCs against anomalous proliferation and apoptosis by inhibiting ERS (endoplasmic reticulum stress) and MAPK signaling pathways induced by mechanical stretch stress—ranging from three major pathways of ER unfolded protein response to its downstream molecular caspase3/12—revealing the potential cardiovascular protective effects of berberine in hypertension (Wang et al., 2020).

Some studies have observed that berberine affected ongoing CVD by inhibiting the intercellular stress of VSMCs. ROS are vital in oxidative stress of VSMCs, and berberine suppressed lysophosphatidylcholine-induced ROS production and the ERK1/2 pathway in VSMCs (Cho et al., 2005). In addition, berberine lowered high blood pressure in mice with deoxycorticosterone acetate–induced hypertension and reduced vascular stiffness in aged ApoE^−/−^ mice by simultaneously acting as an antagonist to the transient receptor potential vanilloid 4 channel, decreasing intracellular calcium levels, and inhibiting calcium overload–caused ERS in VSMCs (Wang et al., 2015). These results show the useful properties of berberine in treating CVDs such as hypertension, vascular stiffness, and stroke.

Berberine also influenced VSMCs to protect the cardiovascular system against complications of other diseases. In a c57/b16 murine model of hypoxia-induced severe pulmonary arterial hypertension, berberine decreased the expression of TGF-β (transforming growth factor-β) and its downstream molecules P-smad2/3 and PPARγ and attenuated related gene transcription, thus inhibiting VSMC proliferation, apoptosis, migration, and extracellular matrix synthesis; consequently, abnormal pulmonary artery vascular remodeling was repressed (Chen et al., 2019). In rats with type 2 diabetes mellitus, berberine alleviated vascular pathological symptoms by lowering the levels of inflammatory cytokines (TGF-β1, IL-6, and TNF-α) in VSMCs (Wu et al., 2020). Matrix metalloproteinase (MMP) has important impacts on VSMCs migration and results in tissue remodeling, which is crucial to the development of arterial maladaptation and stiffness (Belo et al., 2015). Berberine inhibited VSMCs migration in atherosclerosis induced by *C. pneumoniae* infection by downregulating the expressions of MMP3 and MMP9 via suppression of PI3K signaling pathways (Ma et al., 2015). Berberine also inhibited AP-1 and NF-κB signaling pathways, thus reducing the expression of MMP2/9 and u-PA (urokinase-type plasminogen activator) in VSMCs to suppress migration and inflammation (Liu SJ. et al., 2014).

Phenotype switching of VSMCs, also called dedifferentiation, is of great significance in atherosclerosis progression. Synthetic VSMCs have stronger migration and proliferation abilities that usually are accompanied by decreases in expression of smooth muscle-specific markers responsible for vessel contraction and by production of proinflammatory mediators that modulate induction of proliferation and chemotaxis—both of which contribute to vascular remodeling. Many traditional herbs inhibit the phenotype switching of VSMCs in atherosclerosis; examples include chocolate vine, garlic, and mountain asparagus (Saleh Al-Shehabi et al., 2016). Numerous studies have shown that berberine inhibited VSMCs proliferation and migration induced by multiple stimuli (Fatahian et al., 2020; Wu et al., 2020; Zhang et al., 2021), but whether berberine could inhibit the switching of VSMCs from contractile to synthetic phenotypes to reduce atherosclerosis has not been reported and must be explored.

**FIGURE 3:**
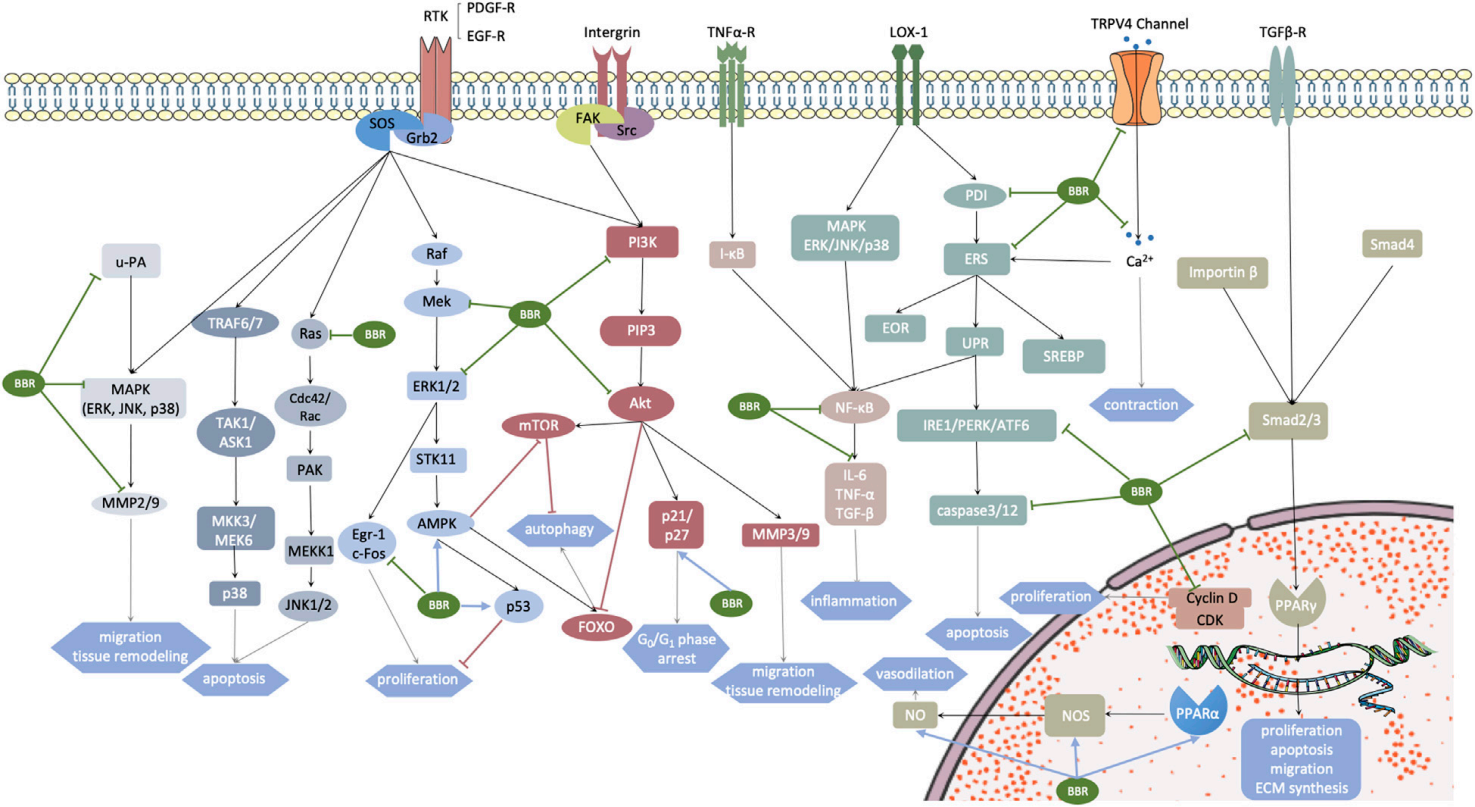
Aberrantly activated signaling pathways in vascular smooth muscle cells (VSMCs) under some pathogenic conditions. Targets of berberine are marked with arrows. The motivated receptors involve transmembrane receptors RTK, integrin, TNF-α, TRPV4 channel, LOX-1, TGF-βR, and nuclear hormone receptor PPARα. These receptors stimulate multiple downstream signaling pathways, promoting a cellular cascade reaction and gene transcription, which subsequently leads to cardiovascular remodeling. Grb2, growth factor receptor-bound protein 2; SOS, Son of Sevenless; u-PA, urokinase-type plasminogen activator; ERK, extracellular regulated protein kinase; JNK, c-Jun N-terminal kinases; P38, P38 MAPK; MMP, matrix metalloproteinase; TRAF6/7, TNF receptor associated factor 6/7; TAK1, transforming growth factor-β-activated kinase 1; ASK1, apoptosis signal-regulating kinase 1; MMK3/MEK6, MAP kinase 3/6; Ras, a family of small GTPase; Cdc42, cell devision control protein 42; PAK, p21-activated kinase; MEKK1, MEK kinase 1; Raf, Raf kinases; Mek, MAPK/ERK kinase 1/2; Egr-1/c-Fos, MEK/ERK-dependent transcription factors, which are related with growth factor activation and cell cycle re-entry; STK11, also known as LTB1, serine-threonine kinase 11; AMPK, AMP-activated protein kinase; PDGF-A, platelet-derived growth factor-A; FAK, focal adhesion kinase; Src, sarcoma; PI3K, phosphoinositide 3-kinases; PIP3, phosphatidylinositol (3,4,5)-trisphosphate; NF-κB, nuclear factor kappa-light-chain-enhancer of activated B cells; IL-6, interleukin-6; TNF-α, tumor necrosis factor-α; TGF-β, transforming growth factor-β; Akt, protein kinase B; Bad, Bcl-2 antagonist of cell death; mTOR and FOXO, two main downstream effectors of AMPK related to cell autophagy; PDI, protein disulfide isomerase; ERS, endoplasmic reticulum stress; EOR, endoplasmic reticulum overload response; UPR, unfolded protein response; PERK, protein kinase R (PKR) like endoplasmic reticulum kinase; IRE1α, inositol-requiring enzyme 1α; ATF6, activating transcription factor 6; SPEBP, sterol regulatory element binding protein; Smad2/3/4, *Drosophila* mothers against decapentaplegic protein 2/3/4; CDK, cyclin-dependent kinase; NOS, NO synthase.

Briefly, the proliferation, migration, apoptosis, and phenotype shifting of VSMCs induce a thickening of the tunica media, which is a dependent event in CVD and the major cause of vascular stenosis. At present, artificial blood vessel graft is applied widely in CVD treatment, but postoperative vascular restenosis caused by VSMCs proliferation remains inevitable. Berberine has shown efficient actions to inhibit both VSMCs proliferation and apoptosis, reflecting its clinical potential (the therapeutic targets of berberine on VSMCs are generalized in Figure 3). However, questions remain: Why do these cells have totally different fates when exposed to the same stimuli? How does berberine inhibit not only proliferation but also apoptosis in VSMCs? Are there other potential targets? Given the importance of phenotype switching of VSMCs in atherosclerosis, mechanism of berberine inhibition of this phenotypic transformation to synthetic VSMCs is unclear. More clinical trials must be conducted to testify the medical effects of berberine on VSMCs.

## Effect of Berberine on Macrophages

Along with constructive cells in the vessel components, macrophages are fundamental distributors in CVDs associated with inflammatory reactions. Their functions include phagocytosing irregular metabolites, modulating inflammatory activity, and producing cytokines and other inflammatory mediators. Berberine suppresses the activity of the mononuclear phagocyte system as one of its anti-inflammatory activities in atherosclerosis. The following subsections will introduce the pro-inflammatory characteristics of macrophages and potential treatment targets of berberine in the process of atherosclerosis.

### Macrophage Functions and Phenotype Change

Activated by chemokines like monocyte chemoattractant protein 1 (MCP-1), monocytes migrate from the blood vessels and differentiate into macrophages. As phagocytic cells and antigen presenting cells, migrative macrophages widely locate in the connective tissues and engulf foreign bodies, such as microorganisms and cell debris mediated by pattern recognition receptor, that are essential participators in innate immunity. In the process of atherogenesis, secretion of various cytokines can regulate the function of the immune response and eventually recruit more inflammatory cells, which enhances monocyte recruitment and foam cell formation.

Resent research has shown that macrophage polarization or phenotype change has a considerable role in the regulation of inflammation. Exposed to different microenvironments, monocytes differentiate into various subtypes of macrophages, including the pro-inflammatory M1 type and the anti-inflammatory M2 type (Liu YC. et al., 2014). M1 macrophages, also known as classically activated macrophages, contribute to an enhanced and persistent inflammatory response by secretion of pro-inflammatory cytokines, for example, IL-6, IL-1β, and TNF-α. Conversely, M2 macrophages induce anti-inflammatory activity and tissue repair activation *via* secretion of anti-inflammatory cytokines such as IL-10, TNF-β, and fibroblast growth factor. Research indicates that modulating macrophage polarization can regulate the atherosclerotic plaque size and stability, which may act as a potential therapeutic approach for atherosclerosis.

### Monocyte Migration and Plaque Formation

Monocyte-derived macrophages are major promoters in the progression of atherosclerosis. In early-stage atherosclerosis, accumulation of low-density lipoproteins (LDLs) in the subendothelial layer enhances the recruitment activation of covering ECs (Mestas and Ley, 2008). Monocytes are attracted to the edge of the endothelial wall by following chemokines. The interaction of endothelial selectins and cooperating ligands mediates the firm adhesion between monocytes and dysfunctional ECs. With chemotaxis, monocytes subsequently migrate through the endothelium and accumulate inside the tunica intima, where they finally differentiate into macrophages under the stimulation of macrophage colony-stimulating factor or other inflammatory factors.

Foam cell formation, which has drawn attention in atherosclerosis research, has considerable significance in the progression of atherosclerotic plaque formation and necrosis. Scavenger receptors, such as CD36 and LOX-1, induce the internalization of LDLs and cholesterol crystals. Reverse cholesterol transporters, such as SR-BI and ATP-binding cassette transporter 1, are beneficial to cholesterol efflux, attenuating the effect of LDL ingestion (Maguire et al., 2019). Macrophages engulf oxLDL and become lipid-loaded cells, or so-called foam cells. Apolipoprotein B accumulation inside macrophages subsequently causes the alteration of macrophage function, thus accelerating the progression of advanced necrotic plaque core formation (Moore and Tabas, 2011). Furthermore, responding to the microenvironment change inside the tunica intima, macrophages adjust their functions *via* phenotype change, which regulates efficiency in the secretion of cytokines and maintains an inflammatory stage in the plaque.

### Berberine Attenuates Macrophage Atherosclerotic Activities

Considerable literature has reported the therapeutic anti-atherosclerosis potential of berberine. Monocyte adhesion and migration activities are initial events in macrophage accumulation under the subendothelial layer. Berberine has effectively reduced the expression of E-selectin and thromboxane B2 (TXB2), weakening the adhesive effect of leukocytes toward ECs, which suggests the possibility of berberine regulation of monocyte recruitment activity induced by endothelium in the development of an inflammatory response (Hu et al., 2009). One study demonstrated that berberine could significantly suppress the secretion of M1-related cytokines, including CXCL16, IL-6, L-selectin, MCP-1, RANTES, and sTNF-R1, in murine splenic macrophages, probably through recruitment of STAT6 expression; this finding indicates a potential therapeutic strategy for berberine in promoting vascular wound healing (Liu YC. et al., 2018). Berberine has also inhibited monocyte mobility during migration by downregulating several infiltration markers. Inhibition of p-p38, p-JNK, nuclear NF-κB, p65, and phospho-p65 by berberine and its derivatives could decrease the expression of macrophage infiltration markers (e.g., CD68, MMP9, and EMMPRIN), reflecting the anti-filtration function of berberine and its derivatives on monocytes (Chen et al., 2014). Moreover, berberine also inhibited macrophage mobility through the participation of toll-like receptors (TLRs) by greatly suppressing Src expression at both the protein and RNA transcription levels (Cheng et al., 2015). The clinical significance of berberine is worth mentioning. In a clinical experiment on acute coronary syndrome, berberine treatment as an adjunct therapy reduced serum levels of key inflammatory markers, including MMP9, ICAM-1, VCAM-1, C-reactive protein, IL-6, and MCP-1, revealing its potential therapeutic anti-inflammatory action in recipients of percutaneous coronary intervention (Meng et al., 2012). Berberine has inhibited vascular inflammation by preventing an increase in ROS production, overexpression of MCP-1, and monocyte adhesion to ECs induced by AngII (Ko et al., 2007).

Macrophage phenotype polarization is due to the microenvironment change and the accumulation of oxLDL. Pro-inflammatory cytokines and chemokines secreted by M1 inflammatory macrophages continuously recruit excess monocytes from the vessel lumen, amplifying the inflammation into a chronic state (Yang et al., 2020). Berberine has inhibited the polarization of pro-inflammatory M1 macrophages by interfering with the TLR4/MyD88/NF-κB signaling pathway and downregulating the secretion of TNF-α and the transcription of inflammatory factors (Gong et al., 2019). An *in vivo* study demonstrated that berberine alleviated inflammatory responses in a murine model of dextran sulfate sodium–induced colitis; mechanistically, the actions were mediated by suppression of M1 macrophage polarization through the Akt1/SOCS1/NF-κB signaling pathway (Liu Y. et al., 2018).

Another study has shown that berberine attenuated foam cell formation, thus regulating the enlargement and rupture of vulnerable plaques. The expression of LOX-1 and CD36 induced by oxLDL was significantly decreased, and the expression of SR-BI was suppressed, with berberine treatment, suggesting the potency of berberine to suppress cholesterol internalization (Guan et al., 2010). Moreover, berberine reportedly suppressed foam cell formation by upregulating the AMPK-SIRT1-PPARγ pathway to weaken oxLDL absorption, and the result was more effective against atherosclerosis when berberine was combined with atorvastatin therapy (Chi et al., 2014). Berberine also inhibited the cholesterol ingestion activity of macrophages and promoted reverse cholesterol transport by upregulating the expression of LOX-1 and decreasing SR-BI expression. In the process of foam cell formation, autophagy reportedly contributes to the facilitation of cholesterol discharge and the enhancement of intracellular lipid droplet hydrolysis in foam cells. Several lines of evidence in current studies have revealed that berberine-induced autophagy controls cholesterol efflux. Animal experiments of insulin resistance have shown that berberine exerts its anti-inflammatory effects through enhanced activation of AMPK-dependent autophagy in adipose tissue macrophages (Zhou et al., 2017)—a finding that may illuminate the study of cholesterol efflux. Another demonstrated that berberine-sonodynamic therapy effectively promoted cholesterol efflux by increasing ROS generation, and this effect subsequently induced autophagy *via* inhibition of the PI3K/Akt/mTOR signaling pathway (Kou et al., 2017). Berberine also induced autophagy in J774A.1-derived macrophages, as mediated by enhanced activation of the AMPK/mTOR signaling pathway, providing its therapeutic potential in treatment of atherosclerosis (Fan et al., 2015).

The majority of studies reported here demonstrated the ability of berberine to suppress monocyte mobility, modulate macrophage phenotype change, and suppress macrophage-derived foam cell formation (Table 2). The study results indicate the therapeutic potential of berberine to counter atherosclerotic plaque formation.

**TABLE 2:** Inflammatory factors and macrophage activity regulated by berberine.

Cell activity	Inflammatory factors or receptors	Possible targeting pathways by Bbr	Effect
Monocyte recruitment	TNF-α, IL-1β	TLR4/MyD88/NF-κB	Downregulate
	IL-6, IL-8	NF-κB and P38	Downregulate
	RANTES, sTNF-R1, L-selectin	STAT6	Downregulate
	TLRs	Src	Downregulate
	MCP-1	STAT6	Downregulate
Monocyte adhesion	E-selectin, TXB_2_	STAT6, AMPK, NF-κB	Downregulate
	ICAM-1, VCAM-1	—	Downregulate
Monocyte migration	MMPs, EMMPRIN	P38, p-JNK, NF-κB p65	Downregulate
Macrophage autophagy	—	PI3K/Akt/mTOR	Downregulate
	—	AMPK/mTOR	Downregulate
Macrophage M1 polarization	TNF-α	TLR4/MyD88/NF-κB	Downregulate
	—	Akt1/SOCS1/NF-κB	Downregulate
Foam cell formation	SR-A	PTEN	Upregulate
	SR-BI	—	Upregulate
	LOX-1, CD36	—	Downregulate

TLR4, toll-like receptor 4; MyD88, myeloid differentiation factor 88; RANTES, regulated upon activation normal T cell expressed and secreted factor; MCP-1, human macrophage chemoattractant protein-1; MMP, matrix metalloproteinase; EMMPRIN, extracellular matrix metalloproteinase inducer; STAT6, signal transducer and activator of transcription 6; PTEN, phosphatase and tensin homolog; SOCS1, suppressor of cytokine signaling 1; SR-A, scavenger receptor class A SR-BI, scavenger receptor class B type I; LOX-1, lectin-like ox-LDL receptor-1.

Given the lipid infiltration and inflammation hypothesis of atherosclerosis, the macrophage-derived foam cell is considered one of the therapeutic sites for lipid metabolism and inflammation-modulating activities. However, some questions and uncertainties require additional exploration in this area. Foam cell formation is related to a series of intracellular processes of oxLDL, including lipid engulfment, cholesterol efflux, and cholesterol ester hydrolysis (Wang et al., 2019). Effect of berberine associated with cholesterol ester hydrolysis in macrophage is still lack of reasearch, which may further determine the therapeutic effect of berberine on foam cell formation. Studies on foam cells mainly focus on scavenger receptors and the cellular activity of cholesterol uptake, whereas the interaction between foam cells and necrotic macrophages is poorly understood. Does foam cell formation exist as an amplification mechanism in the progression of atherosclerotic plaque necrosis? A controversial study showed that berberine could have negative effect on foam cell formation. Another study showed that berberine induced SR-A expression and sustained Akt activation by suppressing PTEN expression in RAW264.7 foam cells at atherosclerotic lesions (Li et al., 2009), which showed that macrophages may increase oxLDL uptake during berberine treatment. This controversial result may counteract the beneficial effect of berberine on other cholesterol receptors and may promote the development of foam cell formation. These results show that additional investigation should be done to evaluate the ability of berberine on various adhesion molecules, cholesterol receptors, and inflammatory factors.

## Effect of Berberine on VSCs

In *Berberine Inhibits EC Dysfunction, Effect of Berberine on VSMCs, Effect of Berberine on Macrophages in Atherosclerosis* sections, we discussed three types of cells that are recognized as having momentous roles in CVD development. Several varieties of stem cells in arterial walls and the circulatory system also play pivotal roles in vascular remodeling and CVD progression (Wang et al., 2018). The stem cells comprise, but are not limited to, hematopoietic stem cells, bone marrow–derived progenitors, mesenchymal stem cells (MSCs), and residential stem cells (Margariti et al., 2006). As adult stem cells, VSCs are quiescent at a normal state; they migrate, divide, or differentiate only after being stimulated by signals from a so-called stem cell niche.

Endothelial injury is the initial incident in the CVD course. Innovative findings show that endothelial progenitor cells (EPCs) might be used in cell therapy for cardiovascular repair despite current controversies (Bianconi et al., 2018). Berberine mobilizes EPCs. Berberine has upregulated the engagement of circulating EPCs, and, as a result, increased small artery elasticity (Xu et al., 2008). Berberine has also activated the PI3K/Akt/eNOS signaling pathway to improve the proliferative capability of EPCs impaired by TNF-α. This finding provides more evidence for EPC cytotherapy in the clinic, especially for diseases that release high levels of TNF-α (e.g., Kawasaki disease) (Xiao et al., 2014). Moreover, the effects of berberine on augmenting circulating EPCs have also been associated with an increasing plasma concentration of NO (Xu et al., 2009). Together, these findings suggest that berberine awakens the potential ability of EPCs to proliferate when the blood vessel is injured or experiences dysfunction.

Another study has shown that berberine safeguards adipose-derived MSCs from apoptosis by decreasing oxidative stress in nutrient-deficient conditions. MSCs are known for their capacity to secrete multiple growth factors; given this property, MSCs have been used in cell therapy. The protective actions of berberine on MSCs are expected to preserve MSCs survival during ischemia (Ghorbani et al., 2018).

Studies of berberine effects on stem cells mainly focus on carcinoma, and research about the effects of berberine on vascular stem cells remains in early stages. What roles does berberine play in VSCs during CVDs progression? An answer to this question requires prospective study results.

## *In Vivo* and Clinical Trials

Hypertension, hyperlipidemia, and hyperglycemia are independent risk factors of CVD. Hypertension can cause cerebral hemorrhage and atherosclerosis; hyperlipidemia increases the risk of vascular embolism; and hyperglycemia directly reflects diabetes. They may exist alone or relate with each other. Many completed or ongoing animal experiments and clinical trials are focusing on the efficacy of berberine in reversing these three risk factors. The studies are summarized in Table 3 and explored in the following subsections.

**TABLE 3:** Berberine in clinical trials for metabolic diseases.

	Study title	Condition or disease	Interventions	Phase	NCT number
Terminated	The Therapeutic Effects of Statins and Berberine on the Hyperlipemia	Dyslipidemias	Drug: Berberine; atorvastatin or rosuvastatin	4	NCT01697735
			Drug: atorvastatin or rosuvastatin		
Completed	Nutraceutical Treatment for Hypercholesterolemia in HIV-infected Patients	Hypercholesterolemia	Dietary Supplement: Nutraceutical combination (NC)	4	NCT03470376
		Inflammation	Behavioral: No nutraceutical combination (noNC)		
		Atherosclerosis			
Completed	A Mechanistic Randomized Controlled Trial on the Cardiovascular Effect of Berberine	Cardiovascular Risk Factor	Drug: Berberine	2,3	NCT03770325
			Drug: Placebo		
Completed	Efficacy and Safety of Berberine in the Treatment of Diabetes With Dyslipidemia	Diabetes Mellitus, Type 2	Drug: Berberine	3	NCT00462046
		Metabolic Syndrome			
Completed	Effectiveness and Safety of Berberine Hydrochloride and Bifidobacterium in People With Abnormal Glucose Level	Berberine Hydrochloride	Drug: Berberine Hydrochloride group	Not Applicable	NCT03330184
			Drug: Bifidobacterium group		
			Drug: Berberine Hydrochloride and Bifidobacterium group		
			Drug: placebo		
Completed	Bioavailability of Berberine and Dihydroberberine and Their Impact on Glycemia	Glycemia	Dietary Supplement: Berberine	Not Applicable	NCT05021341
			Dietary Supplement: Dihydroberberine 200		
			Dietary Supplement: Dihydroberberine 100		
			Dietary Supplement: Placebo		
Completed	Berberine Hyperglycemic Clamp	Diabetes Mellitus	Drug: Berberine Chloride	Early phase 1	NCT03972215
			Drug: Placebo		
Completed	Therapeutic Effects of Berberine in Patients With Type 2 Diabetes	Type 2 Diabetes	Drug: Berberine	1,2	NCT00425009
			Drug: Metformin		
Completed	A Study on the Efficacy and Gut Microbiota of Berberine and Probiotics in Patients With Newly Diagnosed Type 2 Diabetes	Type 2 Diabetes	Drug: 1.Berberine hydrochloride tablets; 2. ProMetS probiotics powder	3	NCT02861261
			Drug: 1. Berberine placebo tablets; 2. ProMetS probiotics powder		
			Drug: 1. Berberine hydrochloride tablets; 2. Probiotics placebo powder		
			Drug: 1. Berberine placebo tablets; 2. Probiotics placebo powder		

A series of completed or terminated clinical trials focusing on the efficacy of berberine in metabolic diseases is summarized in Table 3. A phase 4 clinical trial investigates the therapeutic effects of berberine with or without statins on hyperlipemia patients (NCT01697735). In another phase 4 clinical trial (NCT03470376), investigators tested the effects of nutraceutical combination containing 500 mg berberine to decrease the level of blood lipid and endothelial injury markers among 26 HIV-infected patients. Given that berberine ameliorates many cardiovascular risk factors, a phase 2/3 randomized controlled trial (NCT03770325) assessed the efficacy of berberine in lowering lipid profile, blood pressure, fasting glucose, BMI, etc. Besides, several clinical trials tested the antidiabetic function of berberine on patients with diabetes.

### Berberine and Hyperlipidemia

Berberine has been proven effective in the treatment of various CVDs. A meta-analysis including 27 randomized controlled clinical trials with 2,569 patients demonstrated that berberine had similar therapeutic effects on type 2 diabetes mellitus, hyperlipidemia, and hypertension, with few side effects (Lan et al., 2015). The LDL receptor (LDLR) mediates the endocytosis of LDL, whereas TRIB1 is a GWAS (genome-wide association study) locus closely associated with triglycerides (TGs) and plasma cholesterol levels. Berberine treatment has reduced plasma low-density lipoprotein cholesterol (LDL-C), total cholesterol (TC), and TGs in LDLR-wildtype mice fed a high-fat diet, and it lowered only TGs in LDLR-wildtype mice fed a normal diet. In LDLR-deficient mice (LDLR^−/−^), berberine treatment decreased plasma TG levels by 51% compared with the control group without producing an effect on plasma cholesterol levels (Singh and Liu, 2019). The cholesterol-lowering effect of berberine has also been validated in rats. Rats treated with berberine and an atherogenic diet experienced reductions in the plasma total cholesterol and non-HDL (high-density lipoproteins) cholesterol levels by 29–33% and 31–41%, respectively, possibly as a result of inhibition of intestinal cholesterol absorption (Wang et al., 2014). In another rat experiment, berberine showed a notable lipid-lowering effect, decreasing TG levels by 34.7% and cholesterol levels by 9% (Hu et al., 2012). In hyperlipidemic hamsters, treatment with berberine reduced serum cholesterol by 40% and LDL by 42%. Additionally, berberine has increased hepatic LDLR mRNA by 3.5 times and LDLR protein by 2.6 times (Kong et al., 2004). Combination therapy with berberine and statins also showed good results in lowering lipid levels. In hyperlipidemic rats, the combination of berberine (90 mg/kg/day orally) with simvastatin (6 mg/kg/day orally) decreased serum LDL cholesterol levels by 46.2%; the combination was more effective than berberine alone (26.8%) or simvastatin alone (28.3%) (*p* < 0.01 for both) (Kong et al., 2008).

Numerous clinical trails have tested the effect of berberine in humans as well. Kong et al. (Kong et al., 2004) conducted a clinical trial involving 32 patients with hypercholesterolemia. The results demonstrated that oral administration of berberine for 3 months reduced serum cholesterol by 29%, TGs by 35%, and LDL by 25%, showing that berberine can be used as a novel cholesterol-lowering drug with a mechanism of action that differs from statins. In another clinical trial, obese Caucasian participants were given 500 mg of berberine orally three times per day for 12 weeks. The results indicated that berberine had a mild effect of losing weight (average 5 lb/participant). Interestingly, the lipid levels were reduced, especially the TG levels (23%) and the cholesterol levels (12.2%) (Hu et al., 2012). A meta-analysis involving 11 randomized controlled trials and 1,386 patients demonstrated that, compared with simvastatin, berberine was effective only in lowering TG levels, but showed no statistical significance in LDL and HDL changes. However, the combination of berberine and simvastatin showed better efficacy in reducing TG and total cholesterol levels. Although the results suggested that the lipid-lowering efficacy of berberine is significant, more clinical trials containing larger samples are needed to investigate the optimal therapeutic dose of berberine.

### Berberine and Hypertension

Hypertension is the leading risk factor of CVD. It has been shown in animal experiments and clinical practice that berberine has a hypotensive effect. Luo et al. (Luo et al., 2018) found that berberine attenuated pulmonary arterial hypertension (PAH) both *in vitro* and *in vivo via* the protein phosphatase 2A signaling pathway, so berberine may be an option to treat PAH. In spontaneously hypertensive rats, berberine showed efficacy in lowering blood pressure, preserving arterial elasticity, and improving endothelial function (Zhang G. et al., 2020). These results indicate that berberine is a novel therapeutic option for hypertension. To investigate the clinical effect of berberine in reducing blood pressure and relieving vascular endothelial injury, Tao et al. (2021, NCT04790942, clinic trial) carried out a randomized, double-blind clinical trial of berberine intervention for patients with hypertension. Participants were assigned to the lifestyle intervention group or to the berberine group (berberine tablets 0.4 g, three times per day) for 3 months. The study is currently in recruitment; after 1 and 3 months, participant blood pressure and vascular endothelial function will be evaluated.

### Berberine and Hyperglycemia

Hyperglycemia is the major cause of type 2 diabetes, and it is a risk factor for a series of CVDs. *In vitro* and *in vivo* experiments have validated the efficacy of berberine in the treatment of diabetes and obesity. Berberine reduced body weight and simultaneously improved glucose tolerance in high-fat diet–fed Wistar rats (Lee YS. et al., 2006). Berberine has also facilitated insulin secretion in BALB/C mice, thus reducing blood glucose levels (Leng et al., 2004). Liu et al. (Liu C. et al., 2015) conducted animal experiments of berberine on hamsters fed high-fat and high-glucose (diabetes-inducing) diets, which showed that berberine could reduce glucose levels.

Quite a few clinical investigations of berberine in the treatment of hyperglycemia or diabetes have been reported. Zhang et al. (Zhang et al., 2008) found that fasting and post-load plasma glucose and HbA1c in 59 patients with type 2 diabetes and dyslipidemia were significantly decreased after treatment with berberine (1.0 g daily) for 3 months. The insulin receptor (InsR) is essential for the function of insulin. Low expression of the InsR causes hyperglycemia in mice. As a result, InsR is regarded as a potential therapeutic target in the treatment of type 2 diabetes and insulin resistance. Zhang et al. (Zhang et al., 2010) reported that, when berberine (1.0 g daily) was administrated to 50 patients with type 2 diabetes for 2 months, the proportion of InsR expression by peripheral blood lymphocytes increased significantly. In addition, berberine showed similar efficacy in lowering fasting blood glucose and HbA1c compared with metformin and rosiglitazone, which suggests that berberine is an ideal treatment for type 2 diabetes. During the past decade, the role of gut microbiota in the generation and progression of diabetes and obesity has been widely discussed. In a randomized, double-blind, and placebo-controlled trial with 409 patients with newly diagnosed type 2 diabetes, changes in glycated hemoglobin in groups treated with berberine plus probiotics (used to alter gut microbiota) and berberine alone were distinctly greater than the changes in the probiotics-alone and placebo groups (Zhang Y. et al., 2020). These studies have shown that berberine is useful to treat diabetes, and a combination of berberine and gut microbiota therapy may have additive effects in alleviating hyperglycemia.

## Conclusion and Perspectives

In atherosclerosis, ECs dysfunction initiates the vascular injury, and abnormal VSMCs proliferation, migration, and apoptosis lead to the media thickening; meanwhile, monocytes in the blood cross the endothelial barrier and migrate to the intima to trigger a series of inflammatory responses that contribute to vascular remodeling. Current treatments must improve to prevent disease progression.

Berberine is a natural alkaloid with numerous medicinal properties. Its antioxidant, anti-proliferative, and anti-inflammatory actions inhibit aberrant cell behaviors, such as oxidative stress, proliferation, migration, recruitment, adhesion, and inflammation in CVDs, and have been widely substantiated in cell experiments, animal models, and human studies. Berberine protects ECs from cytokines and inflammatory factors to attenuate vessel remodeling. It also inhibits stimulated VSMCs proliferation to prevent vascular stenosis. Berberine suppresses a series of inflammatory cell activities, such as recruitment, adhesion, secretion, and differentiation, to decelerate vascular inflammation. With regard to VSCs promotion of angiogenesis, berberine mobilizes the proliferating capacity of the endothelial progenitor cell, consequently accelerating its healing process. Berberine also improves mesenchymal stem cells survival in cell therapy.

The current research that both VSMCs proliferation and apoptosis contribute to atherosclerosis explains the new protective effect of berberine against this progress Although angiostenosis has been considered a result of VSMCs proliferation, we found that both proliferation and apoptosis of VSMCs contribute to angiostenosis, providing a new orientation to explore cures for atherosclerosis. Berberine possesses such a dual effect, giving more robust support for its clinical application. Cell therapy in CVD is thriving, and berberine reportedly promotes the survival ability of VSCs, again reflecting its valuable traits.

Although berberine has effective actions against atherosclerosis, its clinical application has progressed slowly and some details remain unknown. Researchers must continue to investigate berberine as its use is developed more in the field of anti-atherosclerosis.
